# Hyaluronic Acid Fillers in Reconstructive Surgery

**DOI:** 10.1111/jocd.16693

**Published:** 2024-11-26

**Authors:** Vespasiani Giordano, Trovato Federica, Ricci Giuseppina, Michelini Simone, Pellacani Giovanni

**Affiliations:** ^1^ Department of Clinical Internal, Anesthesiological and Cardiovascular Sciences, Dermatology Clinic Sapienza University of Rome Rome Italy; ^2^ IDI‐IRCCS Dermatological Research Hospital Rome Italy; ^3^ Dr. Ricci Giuseppina Private Practitioner Rome Italy

**Figure:**
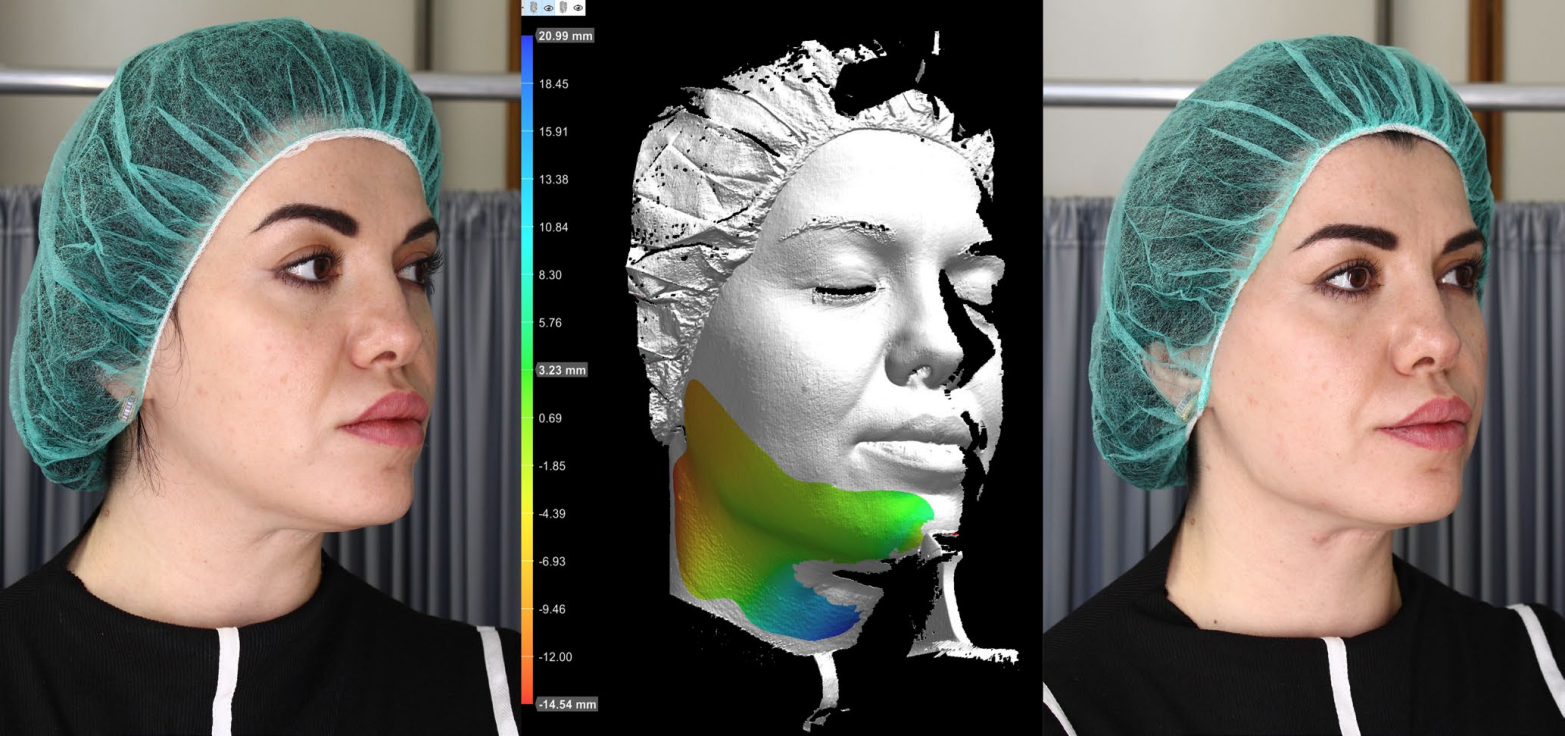
Combo destra

**Figure:**
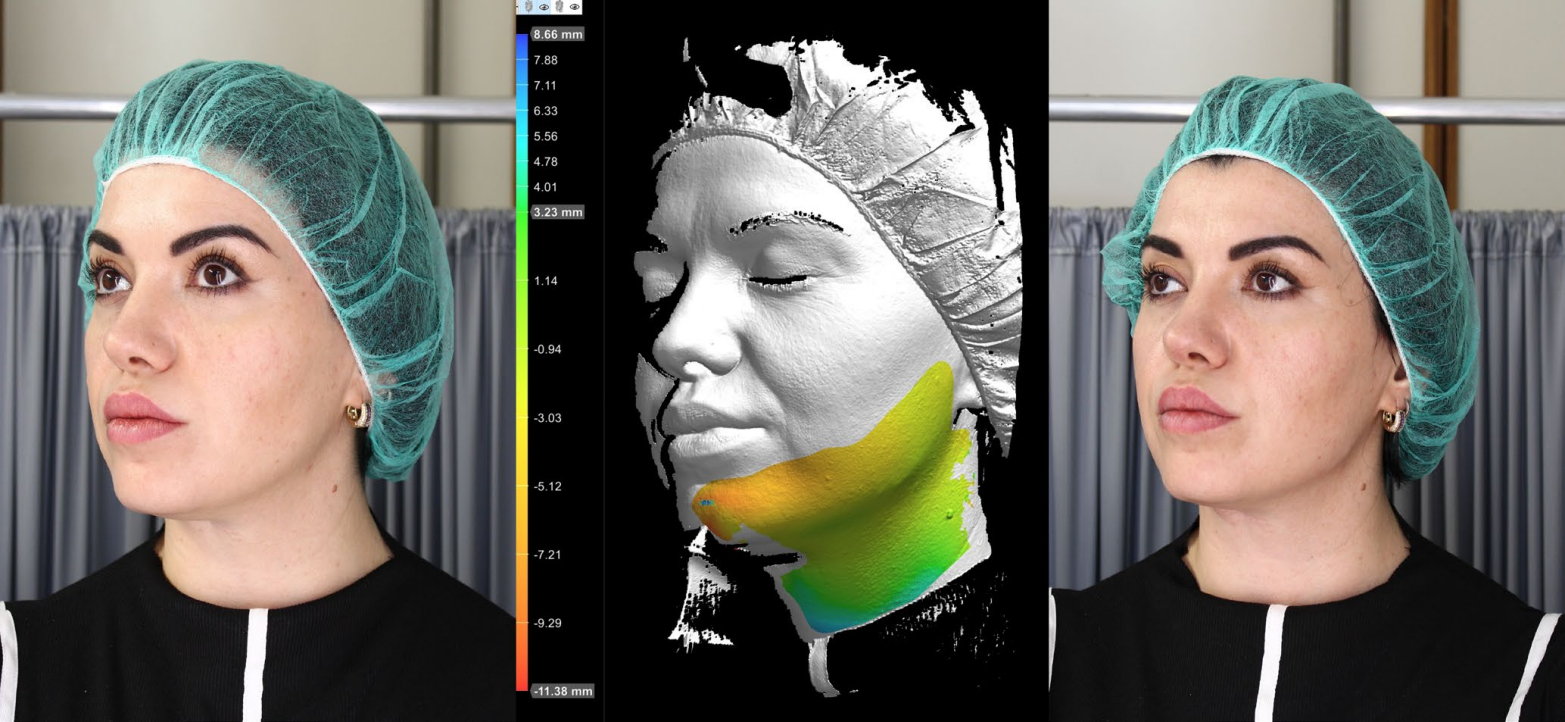
Combo sinistra

Since its introduction, hyaluronic acid (HA) filler has been widely used in medical aesthetics [1]. The main fields of its application are chrono‐aging and wrinkles, facial feminization, lip and buttocks volume enhancement, and improvement of the skin's overall appearance. Occasionally, it has also been used for reconstructive treatments including secondary mastoplasties, secondary rhinoplasties, and secondary cleft lip repairs [2, 3].

Due to its quick resorption rate, HA filler has been scarcely used in reconstructive procedures. At first, HA filler had a maximum 6‐month corrective length, even when cross‐linked with BDDE (1,4‐butanediol diglycidyl ether) [4]. This was a relatively short duration, considering the discomfort that the patient with a mutilation and/or asymmetry would feel.

Advancements in cross‐linking technologies and the discovery of new implantation areas and planes now have paved the way for semi‐permanent corrections, as seen in rhinofiller [5]. New evidences demonstrate the production of new cellular matrix following HA filler stimulation, leading to a progressive improvement in results with repeated sessions [6].

We present a case of a 38‐year‐old female patient self‐referring to our clinic, complaining of an unattractive and unharmonious appearance. Physical examination showed significant asymmetry of the mandibular lines and a paramedian skin depression on the right side of the neck, both caused by a previous excision of the hyoid bone performed 4 years earlier. The patient reported that she had been operated on for a recurrence of a thyroglossal duct cyst, initially treated in 2017, which was causing her neck pain and swelling. The patient was analyzed before and after the procedure using the aesthetic modules of the DermaGraphix software of the CANFIELD V‐track total body videodermoscope, and the AI Vectra software of the CANFIELD VisiaCR genV camera.

Subsequently, we proceeded with the correction of the asymmetric mandibles using HAfiller Techderm Sofiderm DermSubSkin (MCPLE Technology), administered with a 21‐gauge 70‐mm cannula, injecting 1.5 mL of the product: 1 mL in the right retro‐mandibular angle and right hemimandible, and 0.5 mL in the same points in the left hemimandible.

The correction of the pronounced paramedian neck depression was achieved using the same injection technique with 1 mL of HAfiller Sofiderm Derm. The under‐correction obtained on the neck was justified by the patient's expressed complete satisfaction.

The use of AI Vectra applied to VisiaCR allowed us to verify the correctness of the quantity of filler injected, reconfirming VisiaCR as a useful clinical research tool in aesthetic medicine.

The patient was visited in her city 7 months after the procedure. From the clinical evaluation, the correction was still satisfactory, with a reabsorption of about 20% of the injected product.

Our procedural choice was guided by the evidences in literature and the long‐lasting aesthetic corrections achieved with this type of filler, attaining complete patient satisfaction while implementing a procedure with significantly reduced clinical risk and cost compared to surgical intervention. Considering these results, we hope that HA filler will become a routine choice in plastic‐reconstructive procedures in the future.

## Conflicts of Interest

The authors declare no conflicts of interest.

## Data Availability

The data that support the findings of this study are available on request from the corresponding author. The data are not publicly available due to privacy or ethical restrictions.
